# Targeting Epigenetic Mechanisms: A Boon for Cancer Immunotherapy

**DOI:** 10.3390/biomedicines11010169

**Published:** 2023-01-09

**Authors:** Asmita Parab, Lokesh Kumar Bhatt, Abdelwahab Omri

**Affiliations:** 1 Department of Pharmacology, SVKM’s Dr. Bhanuben Nanavati College of Pharmacy, Vile Parle (W), Mumbai 400 056, India; 2 The Novel Drug & Vaccine Delivery Systems Facility, Department of Chemistry and Biochemistry, Laurentian University, Sudbury, ON P3E2C6, Canada

**Keywords:** cancer immunotherapy, epigenetic regulation, tumor microenvironment

## Abstract

Immunotherapy is rapidly emerging as a promising approach against cancer. In the last decade, various immunological mechanisms have been targeted to induce an increase in the immune response against cancer cells. However, despite promising results, many patients show partial response, resistance, or serious toxicities. A promising way to overcome this is the use of immunotherapeutic approaches, in combination with other potential therapeutic approaches. Aberrant epigenetic modifications play an important role in carcinogenesis and its progression, as well as in the functioning of immune cells. Thus, therapeutic approaches targeting aberrant epigenetic mechanisms and the immune response might provide an effective antitumor effect. Further, the recent development of potent epigenetic drugs and immunomodulators gives hope to this combinatorial approach. In this review, we summarize the synergy mechanism between epigenetic therapies and immunotherapy for the treatment of cancer, and discuss recent advancements in the translation of this approach.

## 1. Introduction

Immunotherapy has evolved as a promising therapeutic approach in the treatment of cancer because of its ability to harness the host immune system against tumor cells [1]. It has revolutionized conventional cancer therapy (such as chemotherapy, radiotherapy, and surgery) which aids in improving patients’ survival, especially with metastatic cancers [2]. In cancer, the immune system suppresses tumor growth by eradicating malignant cells, and promotes tumor progression by regulating tumor immunogenicity. This process is commonly referred to as immunoediting which proceeds through three phases elimination, equilibrium, and escape [3]. The innate and adaptive systems initially detect and eliminate the growing tumor cell during the elimination phase. However, a rare tumor variant capable of surviving the elimination phase enters the equilibrium phase, whereby the adaptive system hampers tumor outgrowth and edits tumor immunogenicity. Subsequently, due to constant immune pressure on the tumor cells in the equilibrium phase, cancer cells then bypass the immune system by producing suppressive factors and lose the ability to express the target antigen [3,4,5]. Cancer cells evade immune system attacks through various immune escape mechanisms. This includes immunosuppressive cytokines and immune checkpoint molecules that are expressed on T cells such as programmed cell death 1 (PD-1), programmed death ligand 1 (PD-L1), cytotoxic T- lymphocyte-associated protein 4 (CTLA-4), T cell immunoglobulin and mucin domain 3 (TIM3), and lymphocyte-activation gene 3 (LAG3), are found to be upregulated in cancer and are responsible for tumor immune evasion [6]. Over the past ten years, various immunological mechanisms have been targeted to boost the immune response against cancer cells. The principle behind immunotherapy is to target particular immunological components that control the immune system to regulate the immune system’s natural defenses against cancer cells [7]. Recent developments in cancer immunotherapy have shown tremendous potential, including immune checkpoint blockade, T-cell transfer therapy, and cancer vaccines [6].

**Figure 1:**
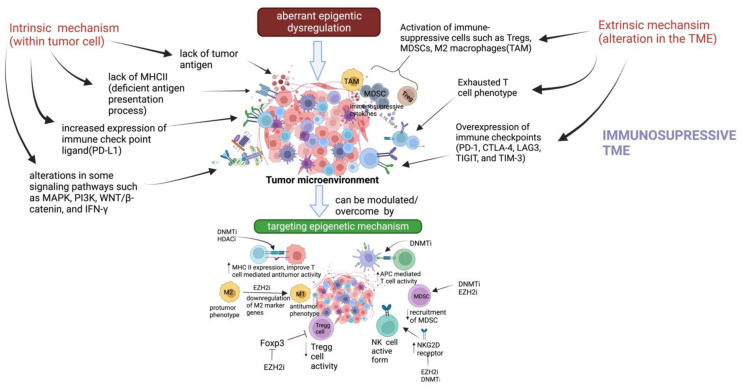
Mechanism of resistance to immunotherapy and combinational approach with epigenetics to improve immunotherapy efficacy: The efficacy of immunotherapy depends on T cell status. The intrinsic mechanism responsible for immunotherapy resistance involves lack of tumor antigen, increase expression of immune checkpoints, lack of MHCII complex, and alteration in signaling pathway; whereas, extrinsic mechanism develops due to alteration in the TME and includes activation of immunosuppressive cells (such as Tregs, MDSCs, M2 macrophage (TAMs) creating an immunosuppressive TME. Aberrant transcriptional programs responsible for cancer initiation and progression are driven by epigenome dysregulation. These epigenomic changes have also been found to modulate tumor immunogenicity and immune cells that participate in antitumor immunity. Hence, targeting epigenetic mechanisms may overcome immunotherapy resistance.

Immune checkpoint molecules are essential inhibitory signaling molecules that control the immune response and prevent autoimmune disorders [8]. These immune checkpoints are found to be upregulated in cancer cells causing them to escape immune attack [9]. So far, antibodies targeting programmed cell death 1 (PD1), PD1 ligand 1 (PD-L1), and cytotoxic T-lymphocyte-associated protein 4 (CTLA-4) are among the FDA-approved ICBs. They block the binding of immune checkpoints to their respective receptor expressed on T cells, and restore suppressed anticancer immunity [2]. Besides ICBs, chimeric antigen receptor-engineered T (CAR-T) cells have been found to be effective in hematological malignancies. CAR-T cells directly act on cancer cells by circumventing general T cell-mediated activity. However, despite promising results, many patients show partial response and resistance to these immunotherapies. The response rate to checkpoint inhibitors and adoptive T-cell therapy significantly varies between different cancer types [10]. The efficacy of immunotherapy depends on T cell status. Immunotherapy resistance can be classified into two types [11,12]: primary resistance and acquired resistance. Primary resistance may be developed due to either the tumor cell (intrinsic resistance) or due to the tumor microenvironment (extrinsic resistance) [10]. Cancer immunoediting is one of the reasons for development of resistance against immunotherapy [4]. The mechanism for low response or resistance to immunotherapy is depicted below (Figure 1). 

A promising way to overcome this is by combining immunotherapeutic approaches with other potential therapeutic approaches which are capable of modulating the cold tumor into a hot tumor [13].

Aberrant transcriptional programs responsible for cancer initiation and progression are driven by epigenome dysregulation. These epigenomic changes have also been found to modulate tumor immunogenicity and immune cells that participate in antitumor immunity [14]. Hence, targeting epigenetic mechanisms in combination with immunotherapy may enhance the efficacy of immunotherapy, and can be a better treatment approach to maximize the benefit of immunotherapy.

## 2. Epigenetic Regulation in Cancer

**Figure 2:**
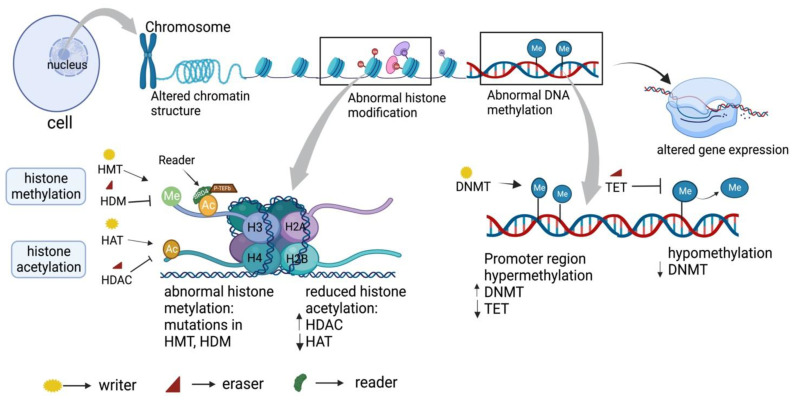
Epigenetic regulation in cancer. DNA within the nucleus of a cell is found to be densely wrapped in the chromatin structure with nucleosome as the basic unit. Alteration in the chromatin structure leads to aberrant gene expression, which is mainly regulated by an epigenetic mechanism. This includes DNA methylation, and histone modification carried out by epigenetic regulators such as writers, erasers, and readers. Histone modification (histone acetylation and histone methylation) maintains chromatin accessibility and gene expression by modulating N and C terminal histone tail. Acetylation of histone, carried by histone acetyltransferase (HAT), is responsible for gene expression, whereas histone deacetylation by histone deacetyl transferase (HDAC) is responsible for gene repression. Similarly, histone methylation, carried by histone methyltransferase (HMT), transfers a methyl group to histone tails from donor S-adenyl methionine (HDM) responsible for gene expression, whereas demethylation by eraser histone demethylase (HDM) resulted in gene repression. DNA methylation by DNA methyltransferase (DNMT) at CpG island, near the promoter region, inhibits gene expression by affecting transcription factor binding to DNA, whereas hypomethylation at the promoter region promotes gene expression. Abnormal DNA methylation and histone modification are observed in cancer generally due to mutation in these epigenetic markers, hence being one of the causes responsible for aberrant gene expression in cancer.

Genetics and epigenetics are two important factors that drive the cancer aggressive phenotype [15]. DNA within the nucleus of a cell is found to be densely wrapped in the chromatin structure, with a nucleosome as the basic unit [16]. Any alteration in the chromatin structure leads to changes in gene expression, an important feature of epigenetic regulation [17]. Epigenetic regulation is a heritable and reversible modification which controls gene expression without affecting the DNA nucleotide sequence [18]. Understanding the origin of epigenetic changes requires an understanding of how chromatin structure is conserved and ordered. Each cell of our body shares the same sets of genes, but functionally differs because of the expression of a specific gene which is regulated by epigenetics [17]. This alteration in gene expression, regulated by the epigenetic mechanism, mainly involved DNA methylation, chromatin remodeling, post-translation histone modification, post-transcription modification of genes such as non-coding RNA (miRNA, lncRNA, circRNA), and others [19]. Epigenetic regulators (writers, erasers, readers) are present near the DNA and the histone protein regulates this mechanism [20]. Writers, such as DNA methyltransferase (DNMT), catalyze DNA methylation, whereas histone acetyltransferase (HATs) and histone methyltransferase (HMTs) carry out post-translational histone modification by binding the N-terminal histone tail. Further readers, such as BET protein, recognize these epigenetic marks, bind to them, and regulate gene transcription by recruiting transcription factor. Epigenetic marks can be further removed by erasers, such as TET (DNA methyltransferase inhibitor), histone deacetylase (HDAC), or demethylase [16,20,21]. Thus, they maintain the chromatin accessibility pattern of gene transcription by controlling the interaction between transcription factor and gene [16,20]. As epigenetic changes regulate gene expression, any dysregulation in epigenetic mechanism results in abnormal gene expression patterns, which are found to be one of the causes of cancer development and progression (e.g., tumor suppressor gene) [5,15]. Mutation across epigenetic regulators is a common cause of mutation observed in cancer [22]. Altered metabolism in cancer promotes tumor growth and survival by modulating epigenetic control of gene expression programs essential for proliferation and adaptive survival [23]. Accordingly, targeted epigenetic therapeutics have been developed for cancer treatment due to the widespread epigenome dysregulation that characterizes human malignancies (Figure 2).

## 3. Epigenetic Regulation of Immune Cells

**Figure 3:**
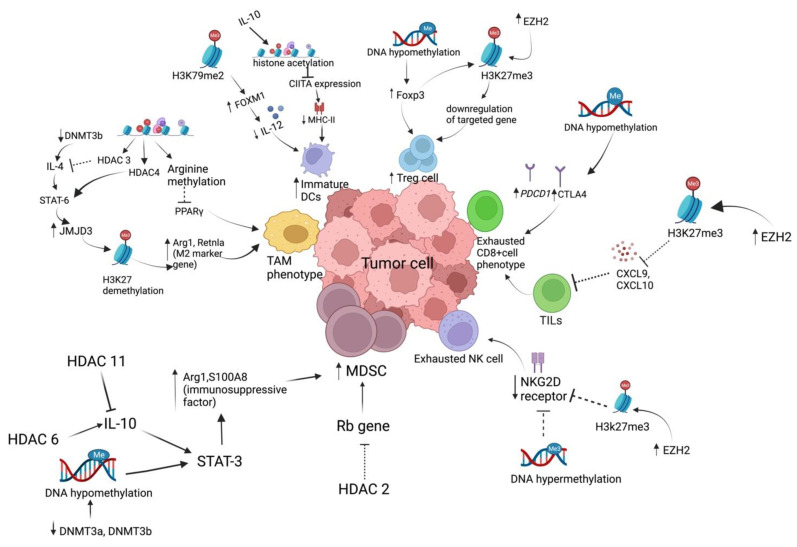
Epigenetic regulation of antitumor immunity. H3K79me2 mediated increased Foxp3 expression associated with decreased production of IL-12, which is important for DC action. IL10-mediated histone acetylation affects type I CIITA expression (positive regulator of MHC-II). Thus, it increases the infiltration of immature DCs in TME. IL4/STAT6 mediated increase in H3K27 demethylation responsible for enhanced expression of M2 marker gene (such as Arg1, Retnla) is associated with M2 macrophage polarization within TME. IL/10/STAT3 mediated Arg1 and S100A8 (immunosuppressive factor) activation is responsible for enhanced accumulation of MDSCs, which is found to be epigenetically controlled by HDAC6, DNMT3a, and DNMT3b. Decreased expression of the activating receptor (NKG2D), associated with exhausted NK cell phenotype, is due to increase in repressive DNA hypermethylation and H3K27me3 state. Overexpressed Foxp3 expression due to hypomethylation state is associated with increased Treg cell accumulation in TME. Dysfunction of cytotoxic T cell in the TME is due to reduced CD8^+^ T cell recruitment into the TME (due to low levels of CXCL9, CXCL10, and CXCL11) and increased exhausted T cell phenotype (characterized by high expression of PD1, TIM3, EOMES, and CD38) regulated by an epigenetic mechanism. H3K27me3 mediated decrease in CXCL9, CXC10 expression is responsible for decrease in T cell infiltration. Increase in DNA hypomethylation state is responsible for enhanced immune checkpoint expression and T cell exhaustion phenotype.

While epigenetic alterations have primarily been studied in relation to cancer cells, mounting evidence suggests that they also play a vital role in modulating the immunosuppressive tumor microenvironment (TME) [24]. The tumor microenvironment (TME) is comprised of immune cells (such as TAMs, DCs, MDSCs, Tregs cells, CD8^+^, CD4^+^, NK cells), stromal cells (fibroblasts), extracellular matrix (cytokines, chemokines, growth factor), and blood vessels present in the vicinity of cancer cells play a crucial role in cancer progression [25,26]. This interplay between cancer and nontumor host cells which drives cancer immune evasion is mainly found to be regulated by aberrant epigenetic mechanisms [27]. Immunological tolerance, developed by TME, is mainly characterized by ineffective T cell activation due to lack of tumor-associated antigens (TAAs), lack of co-stimulatory signals, T cell exhaustion, presence of immunosuppressive cells (MDSCs, Tregg cell), and expression of programmed cell death 1 ligand 1 (PD-L1) [1,5]. Hence, as the epigenetic mechanism regulates this immune cell function of TME, tackling this immunological tolerance by epigenetic modulators may improve the efficacy of immunotherapy. Epigenetic regulation is mainly found to regulate the differentiation and activation of these immune cells. The role of the epigenetic regulation of immune cells mainly macrophages, T cells, and NK cells present within TME, has been discussed below (Figure 3).

### 3.1. Epigenetic Regulation of Myeloid Cells (Differentiation, Activation)

Macrophages, monocytes, and dendritic cells are the most prevalent myeloid cell population found in the TME. They exist in a diverse phenotype which plays an important role in suppressing antitumor immunity [28].

#### 3.1.1. Dendritic Cells

Dendritic cells, commonly known as antigen-presenting cells, serve as a bridge between innate and adaptive immunity, activating T cell-mediated antitumor immunity [29,30,31]. Generally, DCs detect, capture and present tumor-associated antigens (TAAs) produced on cancer cells through MHC expression to cytotoxic T cells by upregulating costimulatory molecules (cytokines, chemokines) which are required for T cell differentiation and activation [5,32]. DCs which are differentiated into many subsets such as conventional DCs (shows stimulatory effect), plasmacytoid DCs (pDCs) (shows tolerogenic effect), and monocyte-derived DCs (moDCs) are present in the TME with different maturation states. The heterogenicity in phenotype and function of these DCs is because of the presence of multiple markers (such as MHC-II, CD80, CD103, etc) due to differences in gene expression which arise during maturation [33]. A high proportion of the immature DCs phenotype compared to the mature DCs phenotype is observed mainly in the TME. These immature TIDCs, abundantly present in the TME, tend to exhibit immune tolerant phenotypes such as low MHC expression, and low co-stimulatory molecule expression [34]. Numerous essential transcription factors (TFs) have been discovered through analysis of gene-targeted mice, this includes Signal Transducer and Activator of Transcription 3 (STAT3), Transcription factor 4 (TCF4), interferon regulatory factor 4 (IRF4), interferon regulatory factor 4 (IRF8), and Kruppel-like factor 4 (KLF4) for DC development [35]. Epigenetic alteration plays an important role in regulating cell differentiation and maturation; hence, it may be associated with this immune-tolerant DCs phenotype.

For example, FOXM1, a proliferation-associated transcription factor, has been found to suppress DC maturation and function in tumor-bearing mice (TBM). Overexpressed FOXM1 was associated with decreased production of IL-12 which is important for DC action. It was seen that upregulated FOXM1 expression in pancreatic and colon cancer was epigenetically regulated by dimethylation on H3 lysine 79 (H3K79me2). Hence, upon inhibition of H3K79 methyltransferase DOT1L, decreased FOXM1 expression with increased IL-12 production was observed. Hence, epigenetically modulated FOXM1 is associated with decreased maturation and function of DCs in the TME [36]. Overexpressed IL-6, present in the TME, also plays an important role in blocking DC maturation via STAT 3 activation [37,38]. This IL-6 expression was found to be regulated by Kruppel-like factor 4 (KLF4) through histone acetylation, as KLF4 levels were found to be downregulated in various types of cancer [39]. Similarly, IL-10 in the TME is also associated with decreased expression of MHC class II on DC, via inhibition of type I CIITA expression (positive regulator of MHC-II) by modulating histone acetylation [40]. Hence, modulation of the DC phenotype by an epigenetic modulator may improve DC-mediated antitumor activity.

#### 3.1.2. Macrophages

Macrophages are essential components of the innate immune system which regulates immunological responses against pathogens, through phagocytosis and antigen presentation [29]. Based on the polarization state, a macrophage present in the TME exists in two phenotype states: classical M1 (pro-inflammatory) and alternative M2 (anti-inflammatory) which inhibit and promote tumor growth, respectively [41]. The differentiation and function of the M1/M2 phenotype are highly influenced by tumor microenvironmental factors (such as hypoxia and cytokines) [42]. The M2 phenotype (also known as Tumor-associated Macrophages) which promotes tumor growth is highly infiltrated in the TME compared to the M1 phenotype (antitumor activity), and is associated with poor prognosis [43]. Epigenetic mechanisms are found to play an important role in controlling this M1/M2 polarization by regulating its key transcription factor [44]. For example, M2 macrophage marker genes (such as Arg1, and Retnla) are epigenetically regulated by H3K27 demethylase JMJD3. It was observed that H3K27, a repressive methylation state present at the promoter locus of the marker gene, was downregulated due to increased JMJD3 expression. IL-4-mediated STAT6 activation plays a crucial role in upregulating this JMJD3 expression [45]. DNMT3b also plays a crucial role in macrophage polarization. Knockdown of DNMT3b was associated with IL-4-induced expression of M2 macrophage markers and vice versa with overexpressed DNMT3b. PPARγ, a key regulator of M2 macrophage, is also regulated by DNMT3b [46]. Similarly, PPARγ gene expression was also found to be regulated by arginine methylation by PRMT1, where knockdown of PRMT1 showed lower PPARγ expression and higher proinflammatory cytokine production [47]. Further histone acetylation has been found to play important role in macrophage polarization: HDAC3 was found to suppress M2 polarization by causing repression of many IL-4-regulated genes [48], whereas, HDAC4 acts as a positive regulator M2 phenotype by inducing STAT6-mediated Arg1 expression [49].

#### 3.1.3. Myeloid-Derived Suppressor Cells (MDSC)

Myeloid-derived suppressor cells (MDSCs) are immature neutrophils and monocytes which are strongly associated with poor clinical outcomes in cancer [50]. MDSCs infiltrated in the TME have been responsible for producing an immunosuppressive TME that facilitates tumor escape. According to mounting data, epigenetic processes influence MDSC accumulation and function.

For example, reduced DNMT3a and DNMT3b expression in THC-induced MDSCs was associated with high expression of STAT3 and Arg1, due to hypomethylation of the STAT3 gene [51]. STAT3-mediated Arg1 and S100A8 (immunosuppressive factor) activation are responsible for the enhanced accumulation of MDSCs in cancer patients [52]. Similarly, histone modification plays an important role in regulating this transcription factor of MDSC. HDAC 11 and HDAC 6 were found to cause repression of the IL-10 gene (STAT3 activator) [53]. Sahakian et al. described HDAC11 as a negative regulator of MDSC expansion [54]. Furthermore, CBP/EP300 bromodomain was also found to regulate MDSC phenotype and expansion in TME through H3K27 acetylation. It was observed that H3K27ac mediated MSDC associated gene (such as Arg1, iNOS, STAT pathway-related genes) expression was downregulated upon inhibition of the CBP/EP300 bromodomain [55]. Polymorphonuclear MDSC (PMN-MDSC) is one of the highly infiltrated MDSC subsets found in many cancer patients [56]. The retinoblastoma gene (*Rb*), a transcriptional regulator, plays an important role in the accumulation of these cells in cancer. Further, Youn et al. showed that silencing of the *Rb* gene, responsible for the accumulation of PMN-MDSC, may be regulated by HDAC2 [57,58].

### 3.2. Epigenetic Regulation of Lymphoid Cells

#### 3.2.1. Natural Killer Cells (NK Cells)

Natural killer cells are one of the important components of innate immunity responsible for causing cytotoxic effects against the tumor. Activation of NK cell cytolytic function is regulated by a variety of germline-encoded receptors which are stimulated by either activating or inhibitory signals [59]. However, in the TME, a limited number of NK cells are found to be infiltrated, and are generally exhausted and functionally impaired [59]. The epigenetic mechanism has been found to regulate the differentiation, maturation, and function of NK cells [60]. NK cell dysfunctional status in tumors is generally associated with decreased expression of the activating receptor (NKG2D, NKp46, KIR2DS) and increased expression of the inhibitory receptor (NKG2A, TIGIT) [61]. DNA methylation and histone modification are mainly found to regulate the transcription of this receptor [62].

For example, the expression of Natural killer cell receptor group 2D (NKG2D), a stimulatory receptor of NK cells, has been regulated by DNA methylation and histone acetylation [63]. Zhao et al. investigated the methylation status of the NKG2D promoter region in hepatitis B and hepatocellular carcinoma (HCC) patients. An increased hypermethylation state was observed in HCC patients, compared to hepatitis [64]. Whereas, high levels of histone H3 lysine 9 acetylation (H3K9Ac) are associated with active transcription of NKG2D receptor, which was proven by HAT inhibitor which showed decreased NKG2D receptor levels [63]. Bugide et al. identified EZH2-mediated H3K27 methylation as a regulator of NKG2D ligands. It was observed that on inhibition of EZH2, NKG2D ligand expression was upregulated, and improved NK cell activity was seen to eradicate HCC [65]. H3K4me3 demethylase Kdm5a was also found to play an important role in the activation of NK cells, as it suppresses H3K4me3 methylation of the Socs1 promoter (a repressor of JAK2-STAT4 signaling pathway) [66].

#### 3.2.2. CD4^+^ T Cells

CD4^+^ T cells part of the adaptive immune system play a crucial role in regulating an antigen-specific immune response against the tumor. Upon activation by antigen-presenting cells, naïve T cells get differentiated into distinct subsets which are mainly influenced by the cytokine milieu of the microenvironment, including T-helper 1 (T_H_1) cells, T-helper 2 (T_H_2) cells, T helper (T_H_17) cells, regulatory T (Treg) cells, follicular helper T cells (T_FH)_, T_H_9 cells [67]. These differentiated CD4^+^ T subsets, characterized by specific transcription factors, exert a broad range of immune responses in TME; T_H_1 (T-bet), T_FH_ (BCL6)_,_ and T_H_9 cells are associated with antitumor activity, while T_H_2 (GATA3), T_H_17 (RORt), T_regg_ (FOXP3) cells promote tumor growth [68]. Epigenetic mechanisms, mainly DNA methylation and histone modification, have been involved in controlling these CD4^+^ T cells differentiation and plasticity by regulating chromatin accessibility of master transcription factors (T-bet, GATA3, RORt, FOXP3) that are in charge of CD4^+^ T cells commitment [69,70,71,72]. For example, loss of EZH2 resulted in a decreased repressive H3K27 methylation state associated with enhanced T_H_1 and T_H_2 cell differentiation and plasticity [70]. Different methylation patterns of CD4^+^ T cells were observed in glioblastoma patients [73]. However, a few functional studies have examined the epigenetic dependence of T helper cells; Treg cells have received the greatest attention when examining the effect of epigenetic modifiers in CD4^+^ T cells.

#### 3.2.3. T_reg_ Cell

FoxP3^+^ Treg cells specialize in immune suppressive responses and are found to be highly infiltrated in the tumor microenvironment associated with poor prognosis [74]. A variety of chemokines (such as CCL28, CCL5) are responsible for Treg cell recruitment at the tumor site [75,76,77]. Foxp3, being the master transcription factor of Treg cells, is epigenetically regulated and is responsible for a stable Treg cell population in tumor-bearing mice and humans. Analysis of Foxp3 revealed a differential methylation pattern between the Treg subset (nTreg and iTreg) and T_conv_ cells. A stable hypomethylation pattern of Foxp3 was observed in the intratumoral Treg cell [78]. Page et al. revealed the crucial role of EZH2 activation, in association with Foxp3, in maintaining the identity of Treg cells after [79]. Foxp3 has been found to act as a repressor upon Treg cell activation, by downregulating the expression of the targeted gene [79,80,81]. Hence, EZH2-mediated repression might be responsible for the suppressive role of Foxp3, because Foxp3-repressed genes are associated with H3K27me3 deposition [79,82]. Inhibition of EZH2 was associated with decreased Treg-mediated immunosuppressive activity in the TME [83].

#### 3.2.4. CD8^+^ T Cell

Upon activation by the antigen, naïve T cells get differentiated into distinct phenotypes, such as effector T (T_EFF_) cells, stem cell memory T (T_SCM_) cells, central memory T (T_CM_) cells, effector memory T (T_EM_) cells, and exhausted T cells which are regulated by epigenetic mechanisms [84]. Generally, dysfunction of the CD8^+^ T cell in the TME is due to decreased infiltration of CD8^+^ T cells into the TME (low levels of CXCL9, CXCL10, and CXCL11) [85,86] and an increased exhausted T cells phenotype (characterized by high expression of PD1, TIM3, EOMES, and CD38) [14,87,88]. Accordingly, epigenetic mechanisms have been found to play an important role in controlling this distinct phenotype in the TME [84].

For example, Peng et al. showed that epigenetic inhibition of DNMT-1 and EZH2 mediated H3K27me3 methylation was associated with improved CD8^+^ T cell infiltration in an ovarian cancer model. It was observed that chemokines such as CXCL9, and CXCL10 required for CD8^+^ T cell recruitment, and were found to be epigenetically repressed by DNMT1 and EZH2 methyltransferase [89]. Genome-wide DNA methylation analysis shows a distinct methylation pattern of tumor-reactive CD8^+^ T cells compared with naïve and effector memory CD8^+^ T cells in colorectal cancer patients. Tumor-reactive CD8^+^ T cells generally showed a methylation pattern like exhausted cells, as exhausted signature genes (*PDCD1* and *CTLA4*) were found to be demethylated the same as how in naïve T cells they were found to be hypermethylated. Similarly, LAG3 (inhibitory receptor) was found to be methylated in naïve cells, but later demethylated in further subsets of T cells [90]. Similarly, histone methylation (H3K4me3 and H3K27me3) has also been shown to regulate CD8^+^ T cell differentiation [91]. Bian et al. showed that H3K79me2 mediated STAT5 expression is associated with CD8 T cells survival, which was dependent on methionine in cancer SLC43A2 [92].

#### 3.2.5. B Cells

Recently B lymphocytes were found to play a critical role in antitumor immunity. There are many different types of B cells present in the TME, including naïve, memory, and terminally differentiated plasma cells [93]. Emerging evidence suggests that the epigenetic mechanism plays an important role in B cell lymphoma by regulating the phenotype of malignant B cells [94].

## 4. Targeting the Epigenetic Mechanism to Modulate Antitumor Immunity: A Strategy to Improve Cancer Immunotherapy

In order to improve the effectiveness of antitumor immunity, the TME characterized by immunosuppressive immune cells should be modulated to immunostimulatory cells. Accordingly, to the above mounting evidence, various epigenetic mechanisms are involved in regulating phenotypes of the immune cells infiltrated in the TME to become immunosuppressive. Hence, modulating this immunosuppressive TME by targeting epigenetic mechanisms by using epidugs (pharmacologic epigenetic modulators) would develop an effective antitumor response (Table 1).

### 4.1. DNMT Inhibitors Targeting Antitumor Immunity

Azacytidine and decitabine are the most successful DNMT inhibitors approved for the treatment of acute myeloid leukemia (AML) and are widely used in clinical trials of cancer immunotherapies [114]. Generally, DNMTi produces a hypomethylating state (permissive state) by either degrading DNMT (nucleotide analogue), or directly binding the methylated region of DNMT (non-nucleotide analogue) [115,116]. DNMTi in cancer treatment were associated with restoring the expression of silenced genes such as tumor suppressor genes, and cancer-associated antigens which were found to be hypermethylated [115,117]. DNMTi were found to regulate immunological signaling in ovarian cancer by enhancing the expression of endogenous retroviral double-stranded RNAs (EVRs), which induced a type I interferon response and stimulated the development of MHC I [95]. It was also observed that increased ERV expression in various cancers was associated with enhanced immune infiltration, higher CD8^+^ T cell infiltration, and improved response to ICB [118]. Hence modulating ERV expression by DNMTi may improve the efficacy of ICB. In addition, there were numerous studies where DNMTi at low doses was shown to improve immunogenicity by enhancing the expression of immune modulatory pathway genes in several human epithelial cancers [115,117]. For example, lower expression of MHC1 (an important antigen-presenting complex), associated with a hypermethylation state, was found to be upregulated by DNMTi demonstrated in various types of cancer models including lung, ovarian, and colon cancer [119,120]. DNMTi were also seen to modulate immunosuppressive cell (such as MDSC and Treg cell) function. For example, decitabine treatment in a leukemia mice model induced an antitumor response by depleting MDSC [97]. Similarly, 5-azacytidine, in myelodysplastic syndrome, was capable of showing an immunomodulatory effect by suppressing the function of Treg cells [96]. Generally, in acute myeloid leukemia, the NK cell’s dysfunctional status associated with decreased expression and function was found to be enhanced by decitabine in combination with adoptive HSPC-NK cells. The combination of decitabine and adoptive HSPC-NK cells resulted in the modulation of NK cell phenotype, activity, and trafficking through upregulation of activating receptors (NKG2D, DNAM-1), inflammatory cytokines (IFN-γ, TNF-α), and perforin [98]. Likewise, the cytotoxic effects of CD8^+^ and helper CD4^+^ T cells were found to be modulated by DNMTi [121]. For example, effector T cell trafficking in the tumor microenvironment, a prerequisite for cytotoxic T cell activity, was found to be improved by DNMTi through upregulation of chemokine (CXCL9 and CXCL10) expression by inhibiting their repressive methylation state [89]. The efficacy of the immune checkpoint blockade was also found to be improved by DNMTi. For example, Ghoneim et al. demonstrated that the combination of DNMTi decitabine with ICB resulted in improved ICB efficacy that restricted ICB responsiveness. DNA methylation was found to play an important role in maintaining the exhaustion-specific gene expression responsible for CD8^+^ cell exhaustion. These DNA-methylation processes, related to exhaustion, were mainly observed in CD8 T cells expressed with high levels of PD-1, hence acting as a barrier to ICB therapy. Therefore, the combination of DNMTi with ICB resulted in the reversal of these programs with an improved T cell response and effective ICB therapy [99]. Furthermore, in murine ovarian cancer, a combination of anti-CTLA4 with decitabine potentiated the efficacy of anti-CTLA4 as it enhanced T cell activity [100].

### 4.2. Tet-2 Role in Modulating Antitumor Immunity

Tet methylcytosine dioxygenase 2 (Tet2) plays an important role in regulating gene expression level by balancing DNA methylation during hematopoiesis, immune cell activation, and immune cell growth [122]. Tet2 is necessary for both the development of cancer and anticancer immune responses, as well as the cross-talk between the immune system and cancer [123]. It is found to enhance the effectiveness of immunotherapy and cancer immunity. For example, Tet2 deletion in mouse melanoma and colon tumor cells was associated with decrease in chemokine expression and tumor-infiltrating lymphocytes [124]. Similarly, Tet-2 deficiency in mice promoted tumor growth due to increased expansion of immunosuppressive granulocytic myeloid-derived suppressor cells (G-MDSCs) [125]. Besides tumor suppressors, inactivation of Tet2 also promoted antitumor activity by enhancing the function of tumor infiltrating lymphocytes [126]. Hence, targeting Tet2 may result in improved antitumor immunity. However, further research is needed to look into how Tet2 interactors can be used to maintain favorable antitumor immune responses and inhibit negative immunological responses to malignancy.

### 4.3. HDAC Inhibitors Targeting Antitumor Immunity

HDAC inhibition in cancer treatment is generally associated with the reactivation of gene expression (i.e., acetylation of histone) that is found to be repressed due to aberrant HDAC expression present in various cancers [127,128]. FDA-approved HDAC inhibitors such as vorinostat, romidepsin, panobinostat, and belinostat are approved for the treatment of certain cancers such as T cell lymphoma and multiple myeloma [129,130]. Besides inducing growth arrest, differentiation, and apoptosis of tumor cells, HDACi were found to modulate tumor immunity. For example, treatment with HDACi Trichostatin (TSA) in metastatic cancer showed increased MHC1 expression on the tumor cell surface, resulting in improved tumor immunogenicity [131]. HDACi is also capable of regulating antitumor immunity by controlling the activity of various dysregulated immune cells in the TME [132]. For instance, Entinostat, a class I HDACi, suppresses regulatory Treg cell function via downregulation of Foxp3 expression in renal and prostate cancer models. Shen et al. suggested that decreased Foxp3 expression was due to STAT3 acetylation (modulator of Foxp3) [101,102]. Furthermore, treatment with Romidepsin was associated with improved NK cell-mediated antitumor immunity in lung cancer due to increased expression of NKG2DL [103]. Li et al., using low-dose HDACi trichostatin, showed enhanced antitumor activity by modulating the immunosuppressive macrophage (M2) phenotype and inhibiting recruitment of MDSC. However, HDAC inhibition also increased PD-L1, which constrained the therapeutic benefits. Compared to the effects of either treatment given alone, the combination of low-dose TSA and anti-PD-1 significantly decreases tumor growth and increases the survival of tumor-bearing mice [104]. HDACi CG-745 in combination with anti-PD1 demonstrated a synergistic anticancer effect by modulating the immunosuppressive tumor microenvironment [105]. Hence, all these data collectively indicate that selective HDACi may be employed as immunomodulatory agents to modulate the suppressive TME, and subsequently enhance ICB therapy.

### 4.4. SIRT-1 [Sirtuin (Silent Mating Type Information Regulation 2 Homolog) 1] Modulating Antitumor Immunity

Class III HDAC SIRT-1, a NAD+-dependent protein deacetylase, was found to play a bifunctional role in tumorigenicity. It was found to suppress, as well as enhance, antitumor immunity [133]. For example, in Limagne et al.’s experiment, SIRT-1 activation by a SIRT agonist inhibited Th17 cell development, which slowed tumor growth in mice via STAT3 deacetylation [134]. Similarly, Ye et al. demonstrated that mesenchymal stem cells (MSC) with overexpressed SIRT-1 exhibited antitumor immunity by increasing the number of CD8^+^ T cells [135]. SIRT-1 activation was also found to inhibit metastasis of hepatocellular carcinoma by inducing M1 macrophage polarization through the NF-B pathway [136]. Therefore, targeting SIRT-1 may enhance antitumor immunity.

### 4.5. HMT Inhibitors Targeting Antitumor Immunity

Besides DNMTi and HDACi, histone methyltransferase inhibitors (HMTi) are also emerging as promising epigenetic therapeutic strategies in cancer immunotherapy. Recently, Tazemetostat, an inhibitor of histone-lysine N-methyltransferase EZH2, received approval for the treatment of metastatic or locally advanced epithelioid sarcoma [137]. Numerous studies have emphasized the importance of the enhancer of zeste homolog 2 (EZH2) in the growth and development of neoplasms, and mutations in EZH2 have been found in a variety of cancers [138,139]. Accumulating studies suggest that EZH2 plays an important role in mediating immune escape by suppressing immune identification and activation, enhancing immunological checkpoints, and fostering an immunosuppressive tumor microenvironment [140]. Several such studies have been investigated using EZH2 inhibitors that demonstrated improved antitumor immunity. For example, in Wang et al.’s experiment, EZH2 inhibition resulted in decreased Foxp3 expression in TI-Treg, and was associated with enhanced CD8^+^ activity [82]. Furthermore, EZH2 inhibition was shown to improve the effectiveness of immunotherapy. For example, EZH2 inhibition enhances antigen presentation and antitumor immunity in head and neck cancer, whereas when treated in an anti-PD1 resistant model, it showed improved efficacy of anti-PD1 therapy [107]. EZH2 inhibition using CPI-1205 improved the efficacy of anti-CTLA-4 therapy. Ipilimumab (anti-CTLA-4) was found to boost EZH2 expression in peripheral T cells. EZH2 inhibition also modulated Treg cell activity via altering Foxp3 expression [106].

### 4.6. PRMT-5 Inhibitor Targeting Antitumor Immunity

Protein arginine methyltransferase (PRMT) mediated histone and non-histone protein methylation plays a crucial role in the development of cancer [141]. Histone arginine methylation by PRMT-5 is generally responsible for either gene activation (H3R2) or gene repression (H4R3), whereas nonhistone arginine methylation is responsible for mRNA splicing, cell cycle processes, and DNA damage responses [142]. Accordingly, to their crucial role in tumorigenesis, PRMT-5 inhibitors (EPZ015666, GSK3326595) have been developed and are in phase 1/2 clinical trials for the treatment of cancer [142]. Besides tumorigenesis, they are also found to modulate antitumor immunity. For instance, the knockdown of PRMT-5 in a cervical cancer model resulted in improved antitumor immunity by reprogramming the T cell-mediated response and regulating PD-L1 expression [108]. Similarly, the combination of a PRMT-5 inhibitor with anti-PD-1 therapy in lung cancer improved antitumor immunity by enhancing the number and function of tumor-infiltrating T cells [109].

### 4.7. Bromodomain Inhibitor Targeting Antitumor Immunity

Bromodomain and extra terminal domain (BET) proteins are commonly known as transcription coregulators and epigenetic readers. They regulate gene expression by recruiting transcription factors upon binding the acetylated lysine histone tail [143]. In cancer, the BRD protein is found to regulate the transcription of various key oncogenes (such as MYC, and CCNA1) [144]. Likewise, several such BETi are in clinical trials for the treatment of cancer. Besides altering tumor cell-intrinsic activity, BETi are also found to control the antitumor immune response.

In syngeneic solid tumor models, RG6146, a non-covalent BETi, was found to improve anti-tumor CD8^+^ T-cell responses. In this study, it was observed that increased tumor cell death caused by RG6146 was dependent on CD8^+^ T-cell-derived tumor necrosis factor (TNF) [110]. Similarly, JQ1, a potent inhibitor of BET, in a syngeneic mouse model was found to promote antitumor immunity by drastically decreasing the expression of PD-L1 on tumor cells, tumor-associated dendritic cells, and macrophages [145]. Another example of JQ1 targeting antitumor immunity and enhancing the efficacy of anti- CTLA4 was in a murine prostate cancer model. The combination of anti-CTLA4 and JQ1 is associated with an enhanced CD8:Treg ratio, and an increase in intratumoral CD8 effector function [146]. Additionally, JQ1 in combination with HDACi promoted T cell-mediated antitumor immunity via suppression of Treg cell function in a lung cancer mouse model [147]. BETi as monotherapy is associated with limited efficacy, due to toxicities and drug resistance [148]. Hence, targeting BET, in combination with immunotherapy or epigenetic therapy in cancer may improve the efficacy of therapy, and thereby prolong the survival of the patient.

## 5. Clinical Trial Combining Epigenetic Therapy with Immunotherapy

Wrangle et al. were the first to report a phase I/II clinical trial on the combination of epidrugs with immunotherapy in patients with non-small cell lung cancer (NSCLC). It was observed that with subsequent immune checkpoint therapy, all five patients who had previously undergone epigenetic therapy reached the 24-week mark without progressing, and three of these experienced high-grade partial RECIST criteria responses that persisted for over 2.5 years in all five patients [111,112].

The combination of immune-checkpoint inhibition and DNMT and/or HDAC inhibition is also being tested in numerous clinical trials for a range of solid tumors, myelodysplastic syndrome, and/or AML which are listed below (Table 2). The main focus of these trials is to check the efficacy of this combination in the treatment of patient populations who are both ICI-resistant and ICI-naive. Notably, many of the trials ongoing are in phase II/III, for example NCT04722952, NCT03094637, NCT02664181, NCT04357873, and NCT04705818. Among current clinical trials, one such study completed was a combination of decitabine with pembrolizumab in patients with AML (NCT02996474). In this trial, a total of 10 participants were enrolled, wherein 6 of 10 patients showed better responses. The study was counted on 21-day cycles. On day 1 of the 21-day cycle, pembrolizumab (200 mg) was administered by IV with decitabine, 20 mg/m squared, by IV on days 8, 12, and days 15 and 19 of every other cycle, and continued for up to 24 weeks (8 cycles). With the exception of three patients who experienced irAEs, the toxicity profile of this innovative combination was mostly consistent with that anticipated when using decitabine alone [113].

## 6. Conclusions

Immunotherapy has emerged as a promising therapeutic strategy in the treatment of cancer. Despite promising results, many patients exhibit a partial response and resistance to immunotherapy. Generally, the efficacy of immunotherapy depends on the immunological tolerance developed by TME, mainly characterized by ineffective T-cell activation due to a lack of tumor-associated antigens (TAAs), lack of co-stimulatory signals, T-cell exhaustion, presence of immunosuppressive cells (MDSCs, Treg cell), and expression of programmed cell death 1 ligand 1 (PD-L1). Combination regimens have the potential to overcome the development of frequently seen, acquired resistance to single-agent immunotherapies. Epigenetic alteration, responsible for cancer initiation and progression, plays an important role in developing this immunosuppressive tumor microenvironment. Several preclinical studies demonstrated that the use of epigenetic modulators enhanced antitumor immunity, and improved the efficacy of immunotherapies when combined. In addition to preclinical studies, early-phase clinical trials on the combinations of epigenetic modulators and immunotherapies have been found to be promising. In the future, acceptance of these methods as standard cancer management techniques will depend on the efficacy results of these trials. Further, identification and validation of patient-specific biomarkers are required, in order to achieve an effective response to the combination. Hence, targeting epigenetic mechanisms in immune and cancer cells will be essential for enhancing antitumor immunity, and creating effective combination therapies for patients with advanced solid tumors.

## Figures and Tables

**Table 1:** Epigenetic modulators targeting antitumor immunity.

Epigenetic Modulator	Clinical Status	Studies Targeting Antitumor Immunity			Reference
		Cancer Model	Type of Immune Cell Modulated	Effects	
**DNMTi**					
		Ovarian cancer cell line		Activate ERVs expression Upregulate immune genes (*IFNβ*, *IRF7*, *STAT3*)	
	Treatment of acute myeloid leukemia and myelodysplastic syndrome	B16-F10 mouse melanoma model	Type 1 Interferon signaling pathway	Enhance immune checkpoint inhibitors (anti-CTLA4) antitumor effects	[95]
Azacytidine		Patient with myelodysplastic syndrome	Treg cell	Increased Foxp3 expression but reduced Treg cell function, producing a large amount of IL-17	[96]
		Leukemia mice model	MDSC	Depletion of MDSC	[97]
Decitabine		Acute myeloid leukemia model	NK cells	Modulation of NK cells phenotype, activity, and trafficking through upregulation of activating receptors (NKG2D, DNAM-1), inflammatory cytokines (IFN-γ, TNF-α), and perforin	[98]
	Treatment of Myelodysplastic syndrome	Ovarian cancer model	CD8^+^ and helper CD4^+^ T cells	Upregulation of chemokines (CXCL9 and CXCL10) expression and increase T cell infiltration in TME	[89]
		Tramp-C2 tumor model	CD8^+^ T cell	Inhibit exhaustion-specific gene expression responsible for CD8^+^ cell exhaustion. In combination with ICB, improve ICB efficacy that restricts ICB-responsiveness	[99]
		Syngeneic murine ovarian cancer model	Nk cells, CD8^+^ T cell	Enhance T cell recruitment and function, and improve the efficacy of anti-CTLA4)	[100]
**HDACi**					
Entinostat	Phase 2 studies in breast and NSCLC cancer showed promising results	Renal and prostate cancer model	Treg cell	Reduced Treg cell function, downregulation of Foxp3 expression due to STAT3 acetylation	[101,102]
Romidepsin	Treatment of cutaneous T-cell lymphoma	Lung cancer cell lines: NCI-H23 and A549	NK cell	Increase expression of NKG2DL, improve NK cell-mediated antitumor immunity	[103]
Trichostatin	Phase 1 study in subjects with relapsed or refractory hematologic malignancies	Syngeneic mouse model	Macrophage (M2) & MDSC	Modulation of M2 phenotype (due to decreased mRNA expression levels of the M2 markers) Increased number of M1 phenotypes within TME. Reduced infiltration of MDSC within TME	[104]
CG-745	Phase II clinical trial pancreatic cancer	Syngeneic mouse model	Macrophage (M2), T cell & MDSC	Suppress M2 macrophage polarization. Effective T cell activity (due to an increase in expression of IL-2 and IFN-γ. Depletion of immunosuppressive cells (MDSC, Treg)	[105]
**HMTi**					
CPI-1205	Phase Ib/II in combination with enzalutamide (E) or abiraterone/prednisone (A/P) in patients with metastatic castration-resistant prostate cancer (mCRPC)	Murine colorectal (M38) tumor model	Treg cell	Reduced Foxp3 expression, Treg cell depletion. Enhanced CD8^+^ activity. Improve the efficacy of anti-CTLA-4 therapy	[83,106]
GSK126 & EPZ6438 (Tazemetostat)	Phase 1 clinical trial in subject with B cell lymphoma, treatment of metastatic or locally advanced epithelioid sarcoma	Human and mouse HNSCC cell lines CAL-33, CAL-27, SCC-9, and SCC-25	Antigen presentation	Upregulation of MHC class I expression	[107]
		PRMT-5i			
EPZ015666	Potent, selective and orally bioavailable PRMT-5 inhibitor	Cervical cancer mice model	CD4^+^ and CD8^+^ T cell	Increased the cytokine secretion from CD4^+^ and CD8^+^ T cells, such as IFN-γ, TNF-α and granzyme B	[108]
GSK591	Chemical probe of PRMT-5 inhibitor	Human and mouse lung cancer cell lines	Tumor-infiltrating T cells	Combination with ani-PD-L1 therapy increases the number and function of tumor-infiltrating T cells	[109]
**BETi**					
RG6146	Early phase clinical trials for the treatment of hematological and solid malignancies	Syngeneic colon and breast tumor model	CD8^+^ T-cell	Enhance anti-tumor CD8^+^ T-cell responses	[110]
JQ1	Tested in clinical trials for a variety of cancers such as NUT midline carcinoma	Syngeneic mouse model	Expression of immune checkpoint	Decreased expression of PD-L1 on tumor cells, tumor-associated dendritic cells, and macrophages	[111]
JQ1 + anti- CTLA4		Prostate cancer model	Antigen processing and immune checkpoint molecules	Increased MHC I expression, decreased PD-L1 expression, increased CD8 infiltration	[112]
JQ1 + HDACi		Murine lung cancer model	Tregg cell	Suppressing Tregg cell function	[113]

**Table 2:** Epigenetic modulator in combination with immunotherapy agent for cancer therapy.

Epigenetic Modulator	Immunotherapy Agent	Condition	Clinical Trial ID	Phase	Status
**DNMT inhibitors**					
Azacytidine	Visilizumab (Anti-CD3 mAb)	Relapsed and refractory acute myeloid leukemia	NCT04722952	III	Recruiting
	Pembrolizumab (Anti-PD1 mAb)	Myelodysplastic Syndrome	NCT03094637	II	Active
	Nivolumab (Anti-PD1 mAb)	Squamous Cell Carcinoma of Head and Neck	NCT05317000	I	Not yet recruiting
	Pembrolizumab (Anti-PD1 mAb)Epacadostat (IDO1 inhibitor)	Solid Tumors Advanced Malignancies Metastatic Cancer	NCT02959437	I/II	Terminated
Decitabine	Nivolumab (Anti-PD1 mAb)	Lung Cancer, Non-small Cell Lung Cancer	NCT02664181	II	Active
	Ipilimumab (Anti-CTLA4 mAb)	Relapsed or Refractory Myelodysplastic Syndrome Acute Myeloid Leukemia	NCT02890329	I	Active
	Pembrolizumab (Anti-PD1 mAb)	Refractory or Relapsed Acute Myeloid Leukemia	NCT02996474	I/II	Completed
Guadecitabine	Atezolizumab (Anti-PD1 mAb), CDX-1401 (cancer vaccine)	Recurrent ovarian, Fallopian tube, or primary peritoneal cancer	NCT03206047	I/II	Active
	Ipilimumab (Anti-CTLA4 mAb)	Metastatic Melanoma	NCT02608437	I	Unknown
**HDAC inhibitors**					
Entinostat	Pembrolizumab (Anti-PD1 mAb)	Myelodysplastic Syndrome	NCT02936752	I	Active
	Ipilimumab (Anti-CTLA4 mAb), Nivolumab (Anti-PD1 mAb)	Breast cancer	NCT02453620	I	Active
vorinostat	Pembrolizumab (Anti-PD1 mAb)	Squamous Cell Lung Cancer, Vulvar Cancer, Penile Cancer, Cervix Cancer, Head, and Neck Squamous Cell Carcinoma, Anal Cancer	NCT04357873	II	Active
Mocetinostat	Durvalumab (IgG1κ mAb)	Advanced or Metastatic Solid Tumors and Non-Small Cell Lung Cancer	NCT02805660	I/II	Terminated
Romidepsin	Nivolumab (Anti-PD1 mAb)	Triple-Negative Breast Cancer	NCT02393794	I/II	Active
DNMTi + HDACi					
Guadecitabine, Mocetinostat	Pembrolizumab (Anti-PD1 mAb)	Lung Cancer	NCT03220477	I	Active
**EZH2 inhibitors**					
Tazemetostat	Durvalumab (IgG1κ mAb)	Advanced Solid Tumor Advanced Colorectal Carcinoma Advanced Soft-tissue Sarcoma Advanced Pancreatic Adenocarcinoma Adult Solid Tumor	NCT04705818	II	Recruiting
	Pembrolizumab (Anti-PD1 mAb)	Advanced Urothelial Carcinoma	NCT03854474	I/II	Recruiting

## Data Availability

No new data were created or analyzed in this study. Data sharing is not applicable to this article.
